# All‐Arthroscopic Muscle Advancement Technique for Repair of Massive Posterosuperior Rotator Cuff Tears in the Lateral Decubitus Position

**DOI:** 10.1002/atn2.70342

**Published:** 2026-11-05

**Authors:** Katsumasa Nakazawa, Tomoya Manaka, Yoshihiro Hirakawa, Yoichi Ito, Rei Nishiura, Suguru Mori, Hidetomi Terai

**Affiliations:** ^1^ Department of Orthopaedic Surgery Osaka Metropolitan University Graduate School of Medicine Osaka Japan; ^2^ Osaka Shoulder Center, Ito Clinic Osaka Japan

## Abstract

Massive posterosuperior rotator cuff tears remain challenging because of the high retear rate after arthroscopic repair. Muscle advancement has been reported as an effective technique for reducing repair tension and improving healing in severely retracted supraspinatus and infraspinatus tendons. Although all‐arthroscopic muscle advancement has recently been reported, previously described techniques were performed in the beach‐chair position. We describe an all‐arthroscopic muscle advancement technique in the lateral decubitus position. This technical note describes the indications, technique, and technical aspects of this procedure. Indications include massive posterosuperior rotator cuff tears in which the supraspinatus and infraspinatus tendons cannot be reduced to the greater tuberosity with conventional release alone, particularly in patients without severe fatty degeneration. The procedure is performed in the lateral decubitus position using a traction device and a 30° arthroscope. Stepwise release of the supraspinatus and infraspinatus from the scapula is performed arthroscopically, followed by rotator cuff repair after sufficient advancement to the greater tuberosity is confirmed. This technique may allow anatomic reduction and low‐tension repair even in difficult‐to‐repair tears. The lateral decubitus position may provide a wider working space and improved visualization around the scapula; however, advanced arthroscopic skills and careful attention to the suprascapular nerve are required.

## Graphical abstract

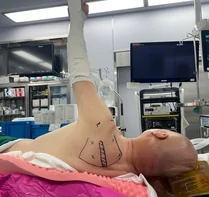

**VIDEO 1:** All‐arthroscopic muscle advancement technique for repairing massive posterosuperior rotator cuff tears in the lateral decubitus position. The patient is placed in the lateral decubitus position using a traction device, and a 30° arthroscope is used throughout the procedure. After intra‐articular release of the capsular attachments beneath the supraspinatus and infraspinatus tendons, tendon mobility is assessed, and stepwise release is performed. The supraspinatus is released from the supraspinous fossa, including the superior angle, superior border, and medial border of the scapula, with careful attention to the suprascapular nerve. The infraspinatus is then released from the infraspinous fossa, including the medial border, inferior angle, and lateral border of the scapula. After sufficient advancement of the supraspinatus and infraspinatus to the greater tuberosity is confirmed, rotator cuff repair is performed using the triple‐row technique. Video content can be viewed at https://doi.org/10.1002/atn2.70342.

According to a systematic review, the retear rate on postoperative magnetic resonance imaging after arthroscopic rotator cuff repair for massive rotator cuff tears remains high at 79%.^1^ Recently, muscle advancement has been reported as an effective method for reducing the retear rate of massive rotator cuff tears. This procedure advances the torn rotator cuff muscles while preserving continuity between their fascial tissue and the rhomboid insertion at the medial border of the scapula, thereby reducing traction tension on the torn rotator cuff. Additionally, the usefulness of all‐arthroscopic muscle advancement has recently been reported. Morihara et al. reported that the postoperative retear rate was <6%, and Gupta et al. reported a rate of 9.3%.^2^, ^3^ Although these techniques were performed in the beach‐chair position, we describe and herein report an all‐arthroscopic muscle advancement technique in the lateral decubitus position.

This study was approved by the Institutional Review Board, and written informed consent was obtained from all patients for the use of clinical photographs, radiographic images, and videos included in this study.

## SURGICAL TECHNIQUE

### Indications for Procedure

All‐arthroscopic muscle advancement is indicated for cases in which the supraspinatus and infraspinatus tendons cannot be reduced to the greater tuberosity footprint with conventional releases. Cases without severe fatty infiltration of the supraspinatus and infraspinatus muscles on magnetic resonance imaging are particularly good candidates. Morihara et al. previously reported that the retear rate increases in cases with Goutallier grade IV rotator cuff muscles. Therefore, we consider cases without advanced arthropathic changes and with supraspinatus and infraspinatus muscles of Goutallier grade ≤3 to be good indications (Figure 1).^4^, ^5^

**FIGURE 1:**
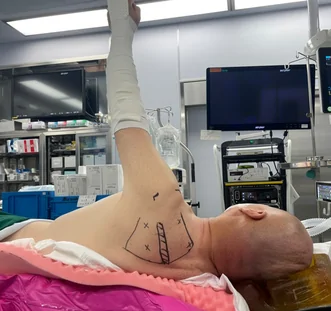
Surgical position. The patient is placed in the lateral decubitus position using a traction device.

### Technique

#### Patient Positioning and Diagnostic Arthroscopy

Patients are placed in the lateral decubitus position using a traction device in the standard manner. A 30° arthroscope is used throughout the procedure (Figure 2). Standard diagnostic arthroscopy is performed through the posterior and anterior portals (Video 1).

**FIGURE 2:**
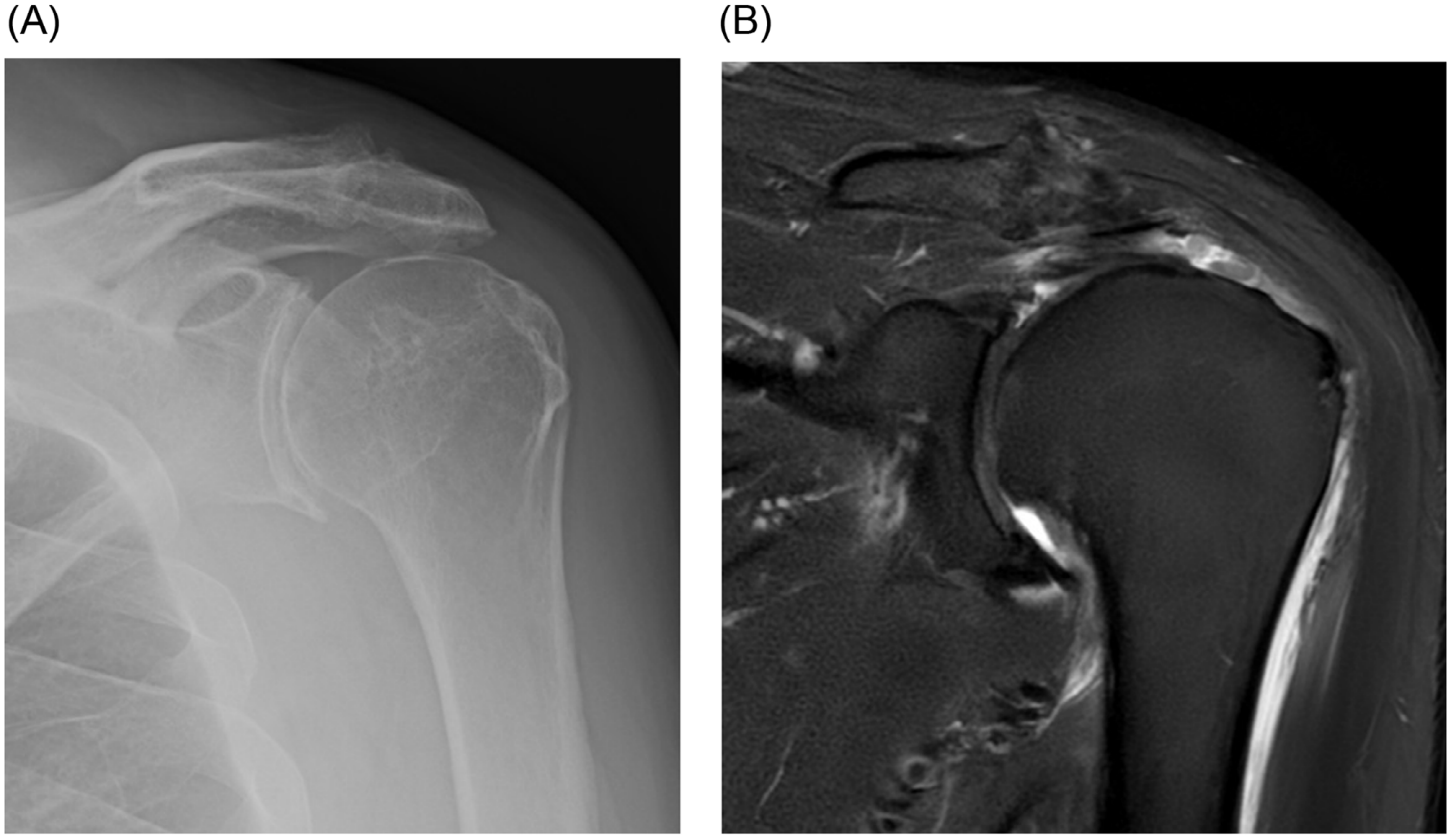
A 56‐year‐old man. (A) Plain radiograph showing arthropathic changes of the glenohumeral joint. (B) Preoperative T2‐weighted coronal MRI showing retraction of the supraspinatus tendon tear to the glenoid level. (MRI, magnetic resonance imaging.)

#### Intra‐articular Release

From the glenohumeral joint, the rotator interval is released using a soft‐tissue shaver and a radiofrequency ablation device alternately. At that time, the coracohumeral ligament is resected. The capsular attachments beneath the supraspinatus and infraspinatus tendons are released from the glenoid. Because the suprascapular nerve is located within the fat pad beneath the inferior slope of the supraglenoid tubercle, care must be taken to avoid nerve injury, and excessive dissection or detachment of the supraglenoid tubercle should therefore be avoided to minimize the risk of iatrogenic suprascapular nerve damage.

The subacromial space is then accessed, and the anterolateral and posterolateral portals are established. Subacromial decompression is performed as needed, and the coracoacromial ligament is resected. The rotator cuff is arthroscopically examined and manipulated to determine reparability. If necessary, concomitant biceps tenodesis is performed.

#### Release and Advancement of the Supraspinatus and Infraspinatus

Four sequential steps are performed according to the degree of tendon retraction (Table 1). The schema of supraspinatus and infraspinatus release during muscle advancement is shown in Figure 3.

**TABLE 1:** Surgical Steps, Pearls, and Pitfalls of Muscle Advancement in the Lateral Decubitus Position

Step 1	Release the capsular attachments beneath the supraspinatus and infraspinatus from the glenoid via the intra‐articular approach
Step 2	Identify the scapular spine
Step 3	Release the supraspinatus from the supraspinous fossa
	Detach it from the superior angle, superior border, and medial border of the scapula
	Special care should be taken to avoid injury to the suprascapular nerve
Step 4	Release the infraspinatus from the infraspinous fossa
	Detach it from the inferior angle, lateral border, and medial border of the scapula

**Pearls**	**Pitfalls**
Select patients with preserved muscle quality and minimal arthropathic change	Forcing the tendon to the greater tuberosity may compromise healing
Perform sufficient circumferential release of the supraspinatus and infraspinatus from the scapula	Poor orientation to scapular anatomy may cause incomplete release or iatrogenic injury
Reassess tendon mobility repeatedly and confirm low‐tension reduction to the footprint	Unnecessary release around the suprascapular notch may cause suprascapular nerve injury
Use scapular landmarks to maintain orientation during release	Persisting with inadequate portals may increase technical difficulty and the risk of soft‐tissue injury
Protect the suprascapular nerve, especially around the suprascapular notch	Prolonged surgery with excessive irrigation may increase the risk of fluid extravasation, airway obstruction, or pneumothorax
Create accessory portals when needed to maintain an appropriate viewing angle and instrument trajectory	Persisting with inadequate portals may increase technical difficulty and the risk of soft‐tissue injury

**FIGURE 3:**
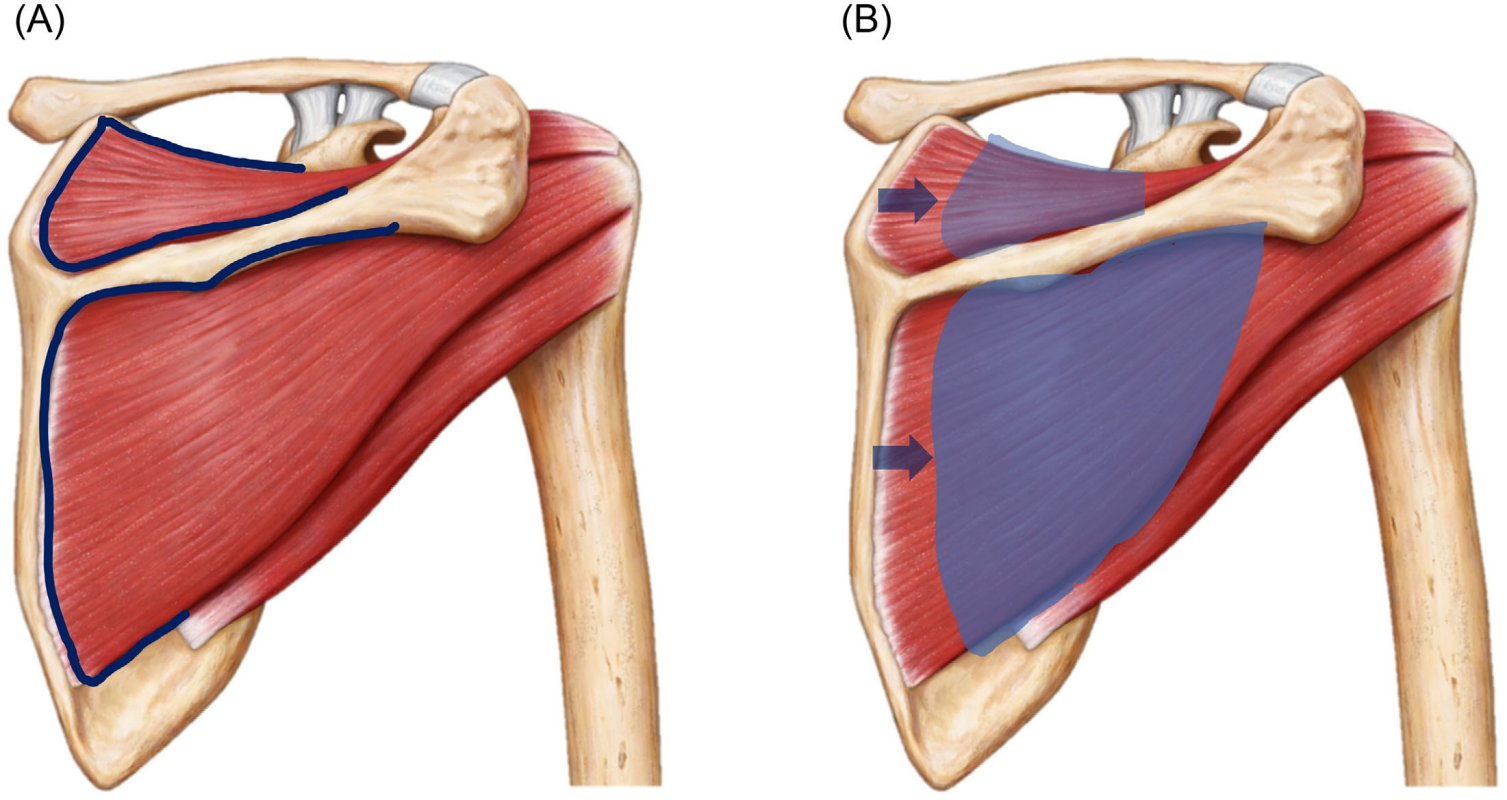
Schema of the muscle advancement procedure. (A) The blue line indicates the release line along the scapular attachments of the supraspinatus and infraspinatus. (B) After release, the muscle is advanced as shown laterally toward the greater tuberosity to permit anatomic, low‐tension rotator cuff repair.

#### Assessment of the Supraspinatus and Infraspinatus Tendons

The supraspinatus and infraspinatus tendons are debrided using a shaver. Next, the coracohumeral ligament is completely released from the coracoid process. Tendon mobility is assessed using an arthroscopic grasper. Stepwise arthroscopic release maneuvers are then performed repeatedly, and tendon mobility is assessed after each step.

#### Release of the Supraspinatus

First, the most lateral edge of the scapular spine is identified. Next, viewing is performed through the posterolateral portal, and working is performed through the anterolateral portal. The supraspinatus muscle is detached from the supraspinous fossa along the scapular spine (Figure 4). If working from the anterolateral portal becomes difficult, the working portal shown in Figure 5 is created. The working portal is created at the midpoint of the line connecting the superior angle of the scapula to the most anterior edge of the acromion. After release to the medial border of the scapula is completed, a viewing portal is created over the superior angle of the scapula (Figure 5). The supraspinatus muscle is then detached from the superior angle and medial border of the scapula (Figure 6). Once the dissection reaches the suprascapular notch, the release is completed. The suprascapular nerve is identified; however, the transverse ligament is not released.

**FIGURE 4:**
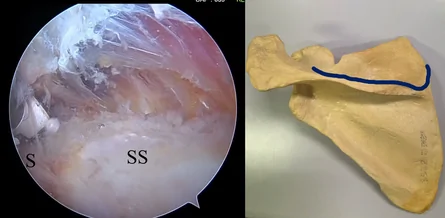
Arthroscopic view of the left shoulder from the posterolateral portal. Working is performed through the anterolateral portal, and the supraspinatus is detached from the scapular spine. Blue line indicates release line. (S, supraspinatus; SS, scapular spine.)

**FIGURE 5:**
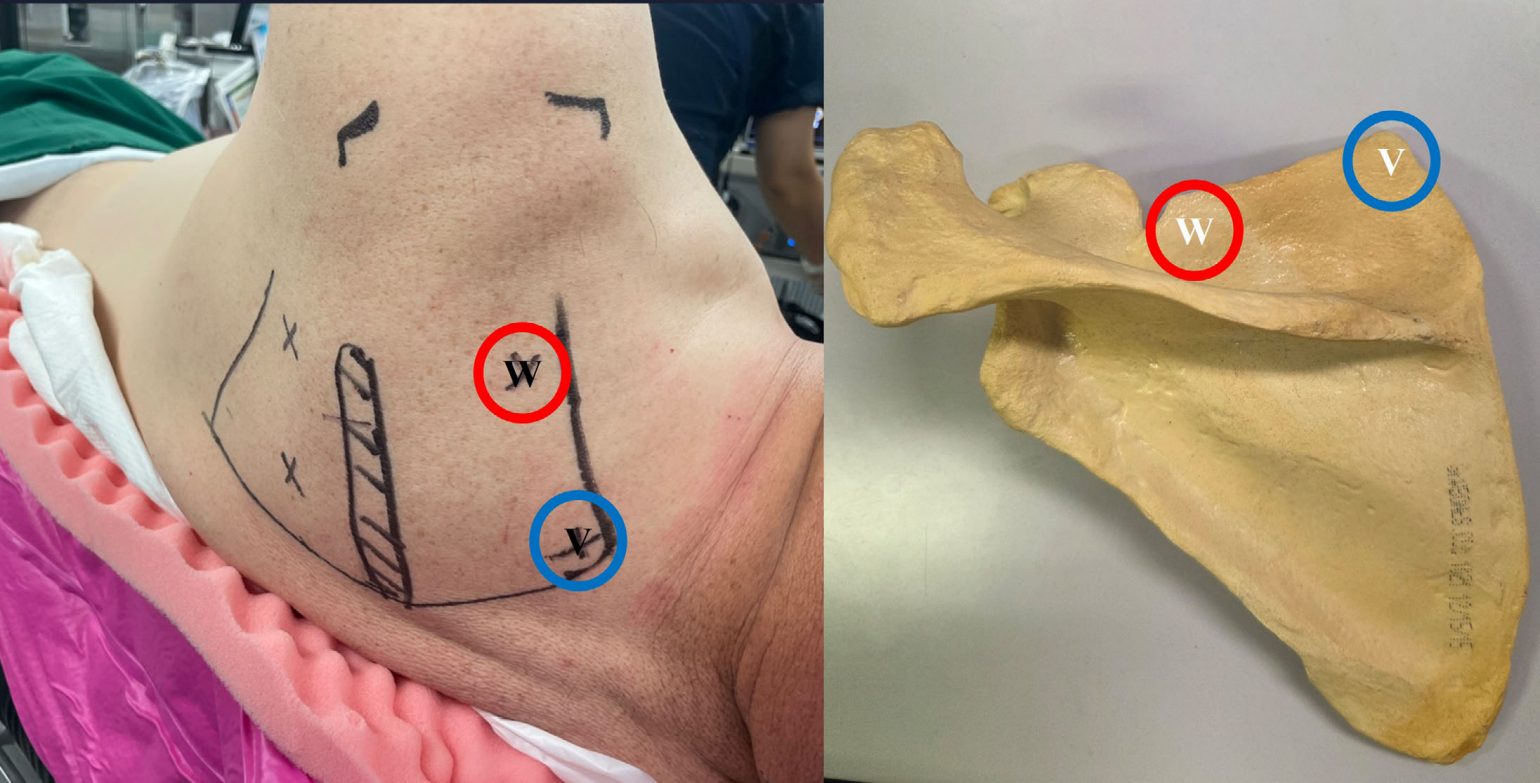
Portals for detaching the supraspinatus from the scapula. The working portal is created at the midpoint of the line connecting the superior angle of the scapula to the most anterior edge of the acromion. After release to the medial border of the scapula is completed, a viewing portal is created over the superior angle of the scapula. (V, viewing portal; W, working portal.)

**FIGURE 6:**
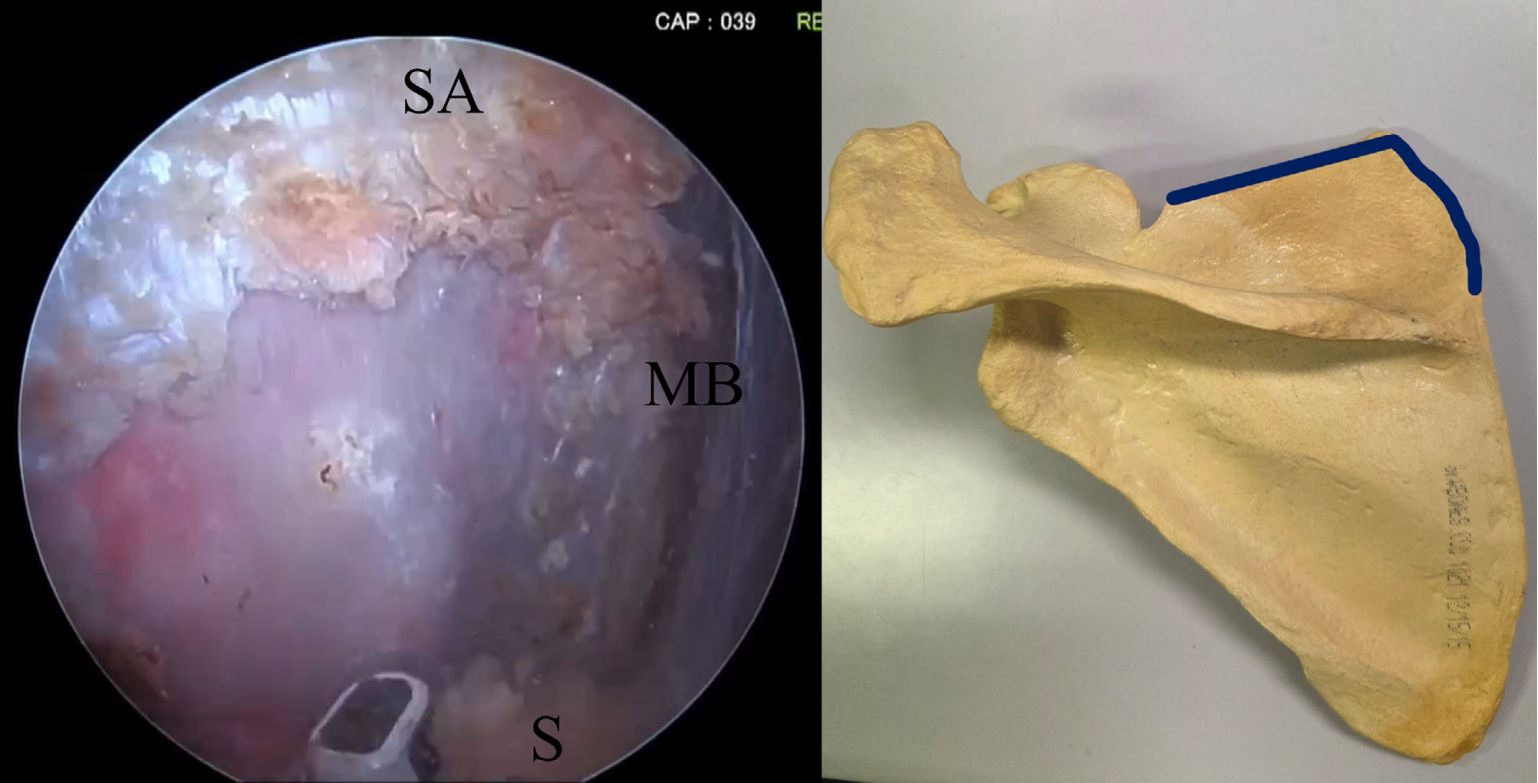
Arthroscopic view of the left shoulder from the viewing portal over the superior angle of the scapula. The supraspinatus is detached from the superior angle and medial border of the scapula, allowing circumferential mobilization of the muscle while maintaining orientation with scapular landmarks and avoiding unnecessary dissection around the suprascapular notch. Blue line indicates release line. (MB, medial border of the scapula; S, supraspinatus; SA, superior angle of the scapula.)

#### Release of the Infraspinatus

Next, the infraspinatus release is performed. Viewing is performed from the posterolateral portal, and working is performed from the posterior portal to release the infraspinatus from the infraspinous fossa along the scapular spine (Figure 7). If posterior portal access becomes difficult, a portal is created as shown in Figure 8. The viewing portal is created near the center of the medial border of the infraspinous fossa, and the working portal is created approximately midway between the posterior and viewing portals (Figure 8). When posterior viewing becomes difficult, the portals are changed, and release is advanced from the medial border, inferior angle, and lateral border of the scapula (Figure 9).

**FIGURE 7:**
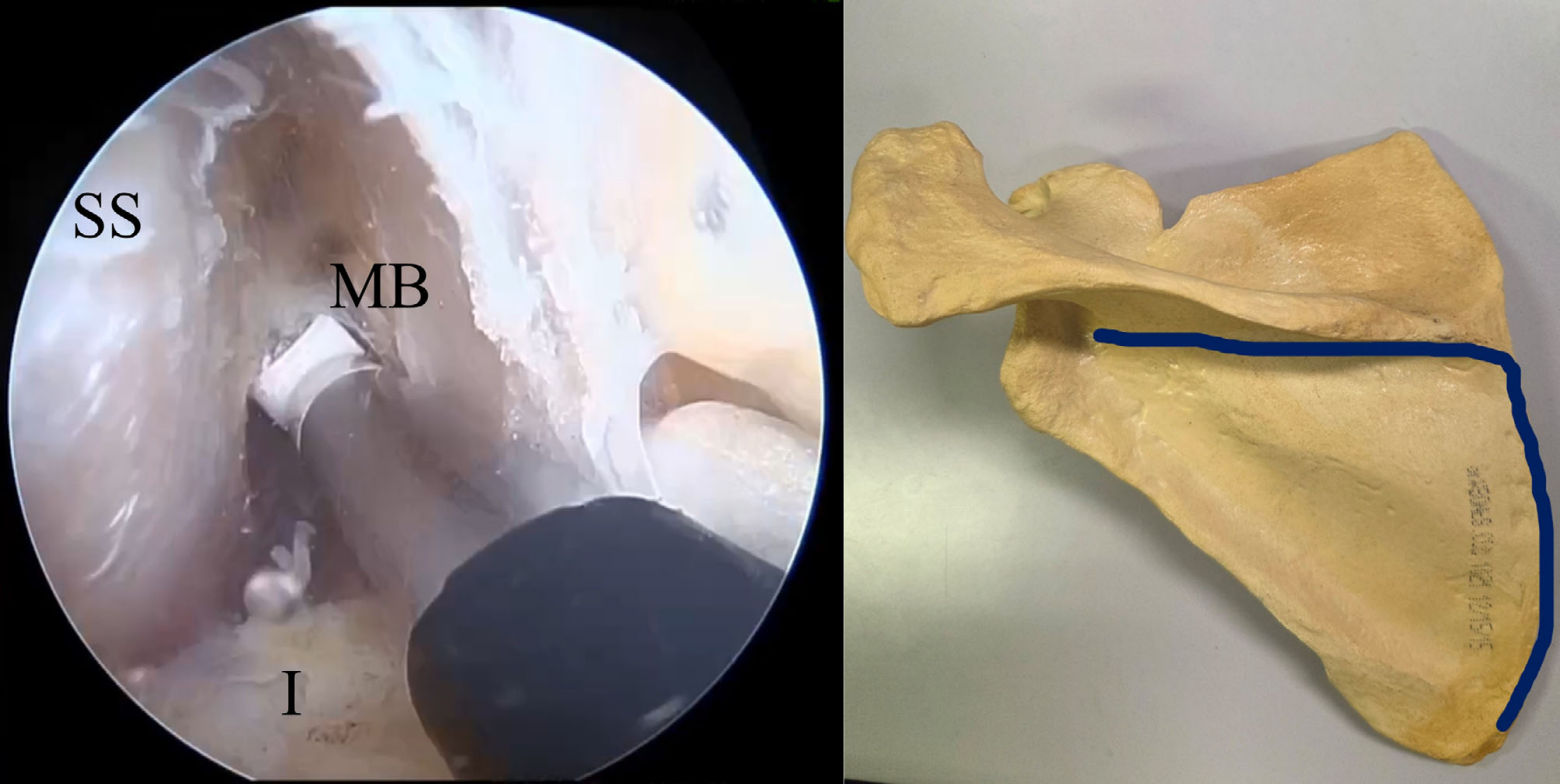
Arthroscopic view of the left shoulder from the posterolateral portal. Working is performed through the anterolateral portal, and the infraspinatus is detached from the scapular spine, extending the release posteriorly to improve lateral advancement of the posterosuperior cuff. Blue line indicates release line. (I, infraspinatus; MB, medial border of the scapula; SS, scapular spine.)

**FIGURE 8:**
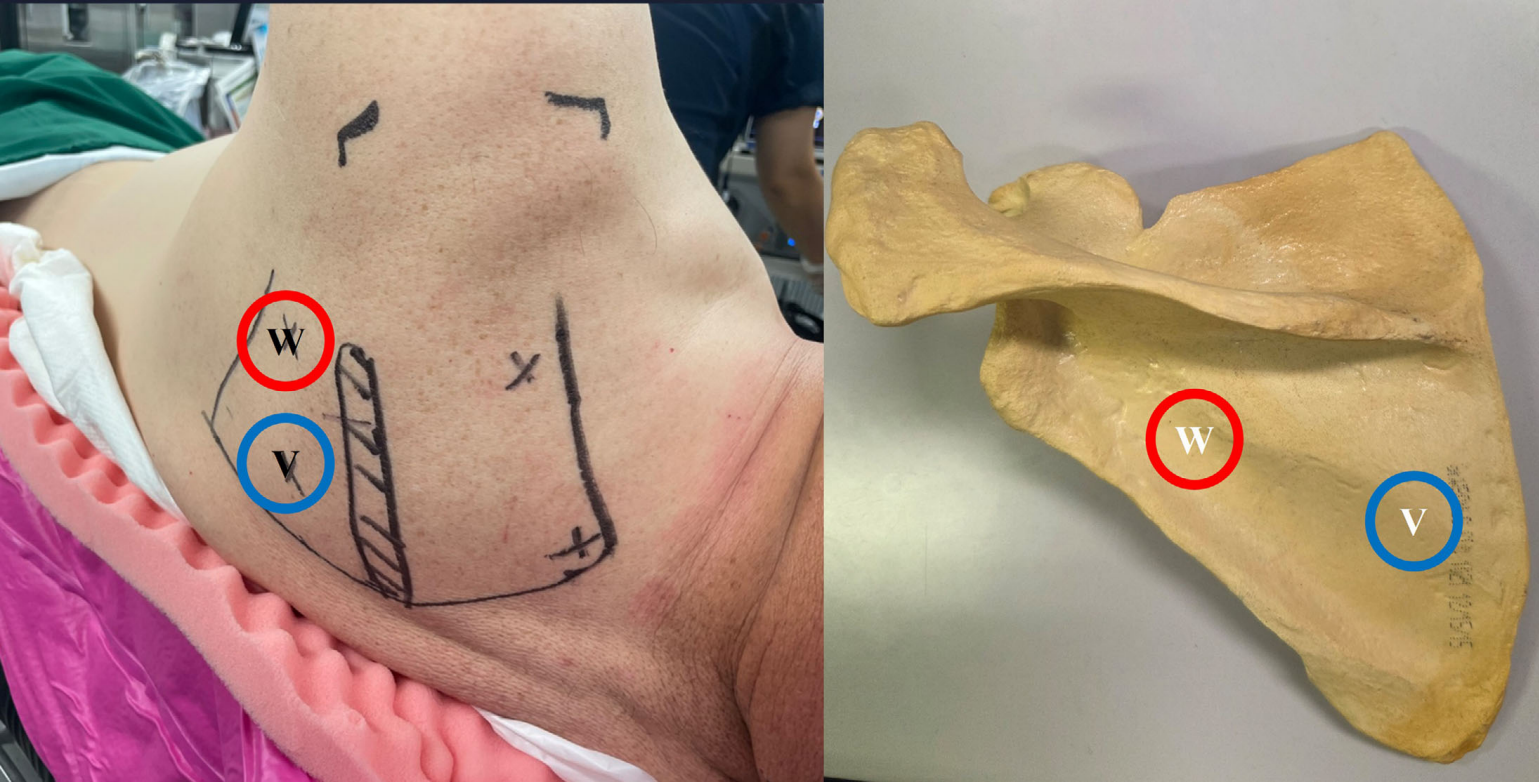
Portals for detaching the infraspinatus from the scapula. The viewing portal is created near the center of the medial border of the infraspinous fossa, and the working portal is created approximately midway between the posterior and viewing portals. (V, viewing portal; W, working portal.)

**FIGURE 9:**
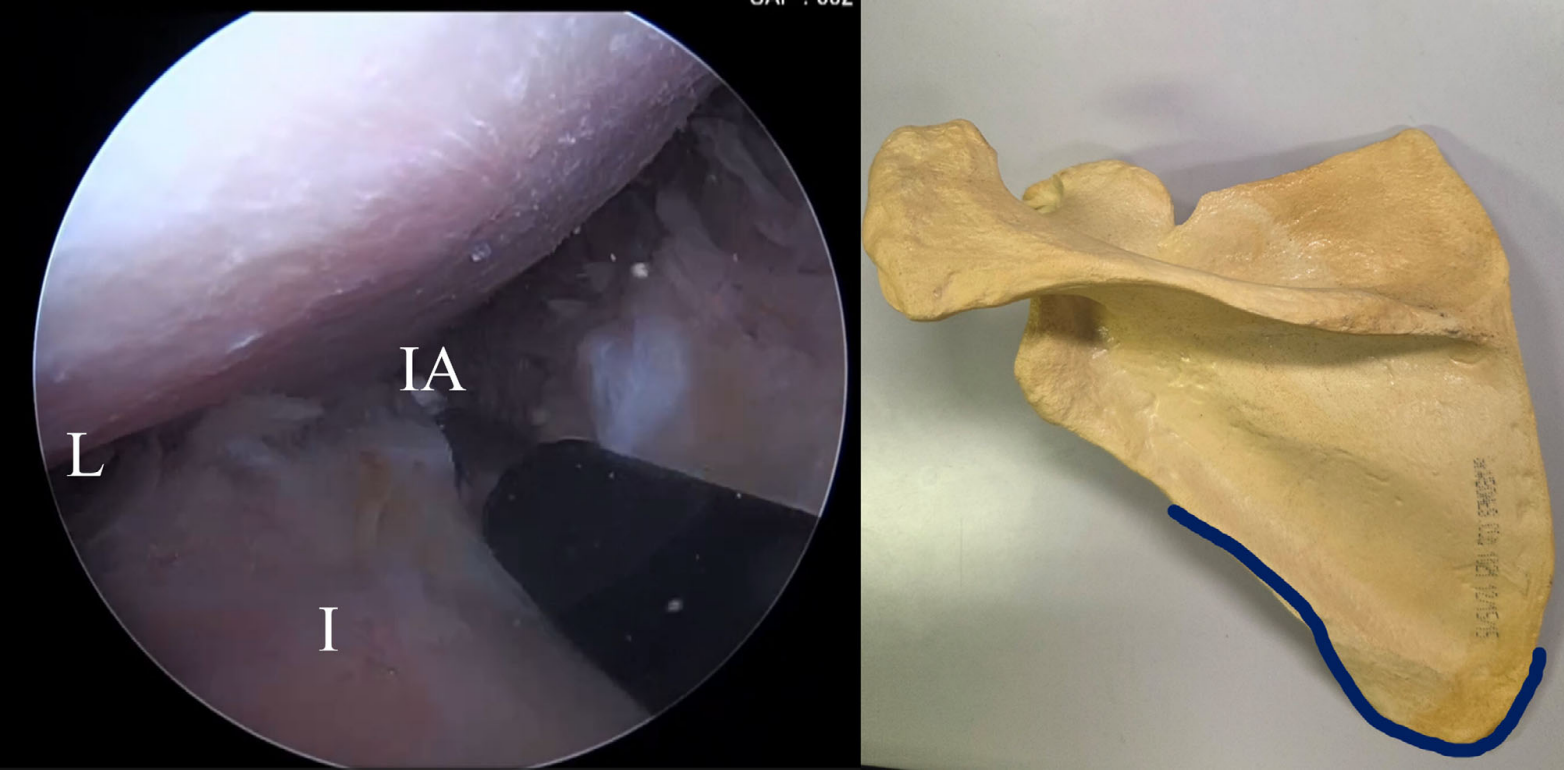
Arthroscopic view from the viewing portal on the infraspinatus side. The infraspinatus is detached from the medial border, inferior angle, and lateral border of the scapula, completing the posterior muscle advancement release and permitting lateral mobilization of the infraspinatus toward the greater tuberosity. Blue line indicates release line. (I, infraspinatus; IA, inferior angle of the scapula; MB, medial border of the scapula.)

#### Rotator Cuff Repair

Rotator cuff repair is performed using the triple‐row technique.^6^ After sufficient release and confirmed advancement of the muscles, 2 or 3 medial‐row anchors are inserted lateral to the humeral articular cartilage (HEALIX ADVANCE BR; DePuy Synthes, Raynham, MA). Two No. 2 permanent sutures are placed approximately 12 to 15 mm medial to the lateral edge of the torn tendon. One or 2 middle‐row anchors are inserted into the greater tuberosity to reduce the torn tendon. One No. 2 permanent suture is passed in a simple fashion. The sutures from the medial anchors are then retrieved, and lateral‐row anchors are inserted into the lateral aspect of the greater tuberosity for fixation (Figure 10).

**FIGURE 10:**
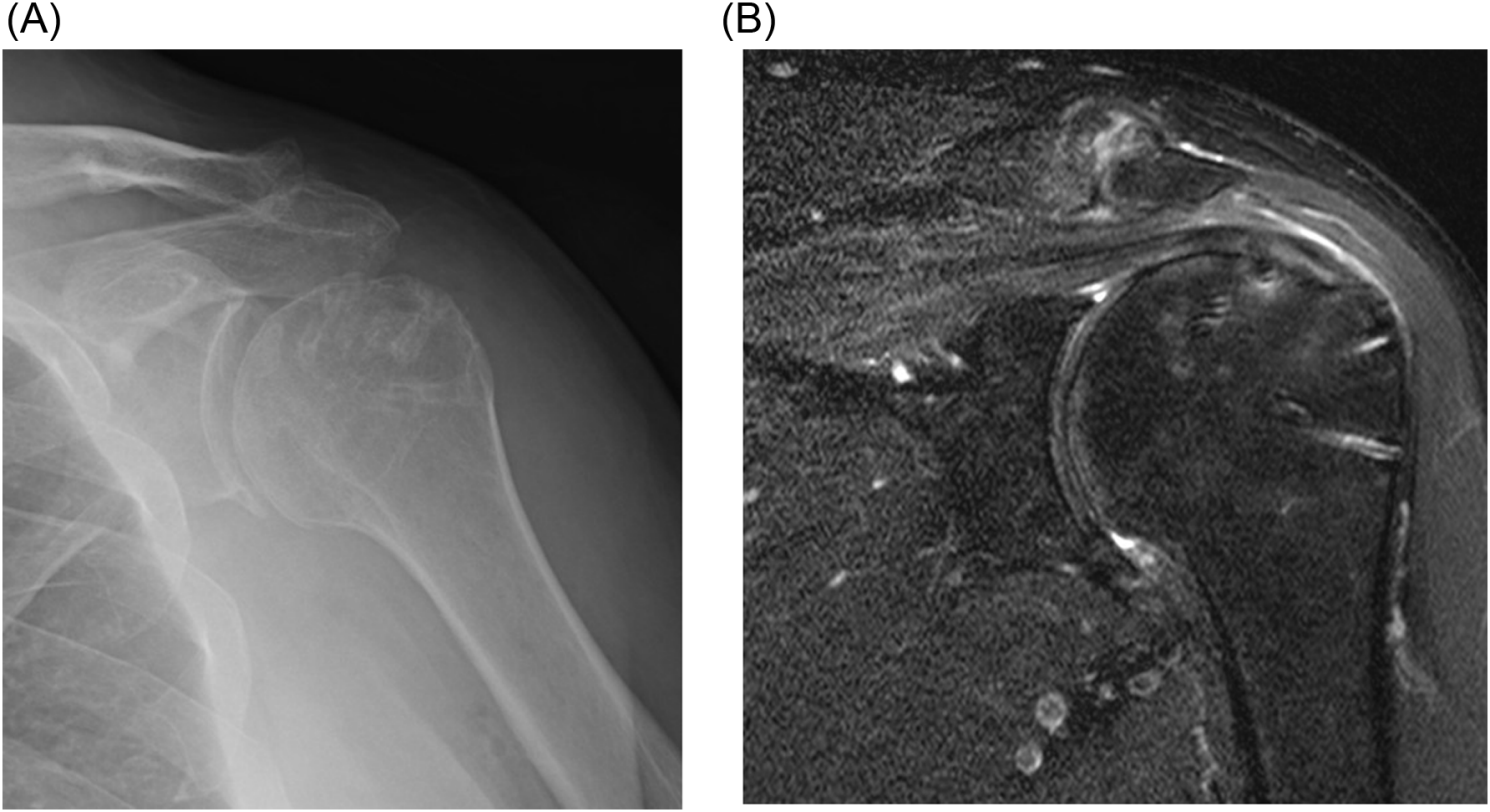
Images obtained 6 months after surgery in the patient shown in Figure 1. (A) Plain radiograph showing no superior migration of the humeral head. (B) T2‐weighted coronal MRI of the right shoulder showing healing of the supraspinatus tendon at its anatomic footprint. (MRI, magnetic resonance imaging.)

### Postoperative Rehabilitation

Patients wear an abduction brace at 30° of abduction for 6 weeks and begin a standardized rotator cuff repair rehabilitation program under the supervision of a physical therapist.

### Institutional Review Board Approval

Approval from the Institutional Review Board of Osaka Metropolitan University was obtained (IRB 2021‐277).

## DISCUSSION

According to a systematic review, the retear rate on postoperative magnetic resonance imaging after arthroscopic rotator cuff repair for massive rotator cuff tears remains high at 79%.^1^ Hein et al. reported that the retear rate was >30% and >40% for rotator cuff tears >3 cm and >5 cm, respectively.^7^ Treatment strategies for irreparable rotator cuff tears include tendon transfer and superior capsular reconstruction. However, the usefulness of muscle advancement for massive supraspinatus and infraspinatus tears has recently been reported.

Muscle advancement was first reported by Debeyre et al. in 1965 as a procedure in which the supraspinatus insertion was released from the scapula, and the muscle was advanced laterally.^8^ Subsequently, arthroscopic‐assisted techniques that completely release the supraspinatus and infraspinatus from their scapular attachments and laterally advance the muscles have been reported. Morihara et al. reported that, after arthroscopic‐assisted muscle advancement, 77% of repairs healed and 23% reteared.^5^ Yokoya et al. reported a retear rate of 23.1%.^9^ More recently, all‐arthroscopic muscle advancement has also been reported. Morihara et al. reported only 1 retear among 18 shoulders, and Gupta et al. reported 4 retears among 43 shoulders (9.3%).^2^, ^3^ Morihara et al. reported that the lower retear rate with the all‐arthroscopic technique compared with the arthroscopic‐assisted technique was because of precise circumferential release of the supraspinatus muscle from the superior angle to the medial border of the scapula, including the superior border, thereby permitting low‐tension advancement.^3^ Gupta et al. also reported that the muscle advancement technique increased lateral excursion of the torn tendon by 4 to 5 cm.^10^ As a result, the rotator cuff can be repaired in a tension‐free state, which may improve healing rates. However, these reports of all‐arthroscopic muscle advancement were performed in the beach‐chair position, and no studies have reported outcomes in the lateral decubitus position.

Therefore, we adapted Morihara's all‐arthroscopic procedure for use in the lateral decubitus position and describe the modified technique herein. The advantages of this position include enlargement of the subacromial working space with traction, resulting in easier visualization and instrument handling.^11^

Additionally, compared with the beach‐chair position, the scapula is more fully exposed, which may facilitate scapular‐side procedures. However, disadvantages include the need to adapt to a nonanatomic spatial orientation and increased difficulty in performing intraoperative shoulder rotation maneuvers compared with the beach‐chair position.^11^ It may also be more difficult for assistants to visualize the operative field. However, we believe that all‐arthroscopic muscle advancement in the lateral decubitus position enables sufficient release of the supraspinatus and infraspinatus from the scapula, similar to the beach‐chair position, and allows reproducible low‐tension rotator cuff repair.

However, this technique has several limitations. First, it requires advanced arthroscopic skills and detailed anatomical knowledge. Additionally, even among massive rotator cuff tears, Morihara et al. previously reported that the retear rate increases in cases with Goutallier grade 4 rotator cuff muscles; therefore, cases with grade ≤3 are considered indicated, whereas cases with severe arthropathic changes are considered contraindicated.^5^ Furthermore, possible complications include scapular fracture and suprascapular nerve injury; additionally, in the lateral decubitus position, hydrothorax‐related spontaneous pneumothorax and airway obstruction caused by irrigation fluid.^12^

This technique allows anatomic reduction and tension‐free repair of the rotator cuff, even in the lateral decubitus position, and may reduce the retear rate without graft use in patients with tears not amenable to primary repair (Table 2).

**TABLE 2:** Advantages and Disadvantages of Performing All‐Arthroscopic Muscle Advancement

**Advantages**	**Disadvantages**
Completely arthroscopic procedure	Requires advanced arthroscopic surgical skills
No graft required	Requires advanced knowledge of shoulder anatomy
Anatomic repair may improve the healing potential	Risk of suprascapular nerve injury
Tension‐free repair	Risk of scapular fracture
Enables precise circumferential muscle release	Risk of spontaneous pneumothorax or airway obstruction because of irrigation fluid extravasation
Traction increases subacromial working space, facilitating visualization and instrument handling	Contraindicated severe osteoarthritic change or Goutallier grade ≥4 muscle atrophy
Improved scapular exposure facilitates surgical manipulation compared with the beach‐chair position	

We believe that this all‐arthroscopic muscle advancement technique in the lateral decubitus position enables anatomic reduction and tension‐free repair of the rotator cuff and is useful for difficult‐to‐repair cases.

## DISCLOSURES

The authors (K.N., T.M., Y.H., Y.I., R.N., S.M., H.T.) declare that they have no known competing financial interests or personal relationships that could have appeared to influence the work reported in this article.
